# Maternal safety of the delayed-release doxylamine and pyridoxine combination for nausea and vomiting of pregnancy; a randomized placebo controlled trial

**DOI:** 10.1186/s12884-015-0488-1

**Published:** 2015-03-18

**Authors:** Gideon Koren, Shannon Clark, Gary D V Hankins, Steve N Caritis, Jason G Umans, Menachem Miodovnik, Donald R Mattison, Ilan Matok

**Affiliations:** From the Motherisk Program, Hospital for Sick Children and University of Toronto, Toronto, Canada; Department of Obstetrics and Gynecology, University of Texas, Medical Branch Galveston, Galveston, TX USA; Department of Obstetrics and Gynecology, University of Pittsburgh, Medical Center, Pittsburgh, PA USA; Medstar Health Research Institute, Hyattsville MD, and the Georgetown- Howard Universities Center for Clinical and Translational Science, Washington, DC USA; The Obstetric Pharmacology Research Unit Network, Eunice Kennedy Shriver, National Institute of Child and Human Development, Bethesda, MD USA; Division of Clinical Pharmacy, Institute of Drug Research, School of Pharmacy, Faculty of Medicine, The Hebrew University of Jerusalem, Jerusalem, Israel; Division of Clinical Pharmacology/Toxicology, Hospital for Sick Children, 555 University Avenue, Toronto, ON M5G 1X8 Canada

**Keywords:** Doxylamine, Pyridoxine, Nausea and vomiting of pregnancy, Diclegis, Safety, Toxicology

## Abstract

**Background:**

Nausea and vomiting of pregnancy (NVP) is the most common medical condition in pregnancy, affecting up to 80% of expecting mothers. In April 2013 the FDA approved the delayed release combination of doxylamine succinate and -pyridoxine hydrochloride (Diclegis®) for NVP, following a phase 3 randomized trial in pregnant women. The fetal safety of this medication has been proven by numerous studies. However, because it is the only FDA-approved medication for NVP that is likely to be used by a large number of pregnant women, its maternal safety is an important public health question. The Objective is to evaluate the maternal safety of doxylamine succinate -pyridoxine hydrochloride delayed-release preparation (Diclegis® as compared to placebo.

**Methods:**

We randomized women suffering from NVP to receive Diclegis® (n = 131) or placebo (n = 125) for 14 days at doses ranging from 2–4 tablets a day, based on a pre-specified titration protocol response to symptoms. Adverse events were collected through patient diaries, clinical examination and laboratory testing.

**Results:**

Doxylamine succinate 10 mg and pyridoxine hydrochloride 10 mg use was not associated with an increased rate of any adverse event over placebo, including CNS depression, gastrointestinal or cardiovascular involvement.

**Conclusions:**

Doxylamine succinate–pyridoxine hydrochloride delayed release combination is safe and well tolerated by pregnant women when used in the recommended dose of up to 4 tablets daily in treating nausea and vomiting of pregnancy.

**Trial Registration:**

Clinical Trial Registration No: NCT00614445.

## Background

Nausea and vomiting of pregnancy (NVP) affect up to 80% of expecting mothers, and while lifestyle changes may be helpful, many women need medications to control their symptoms [1,2].

The delayed-release combination of doxylamine succinate and pyridoxine hydrochloride (Bendectin®) was commonly used for NVP and was the only drug approved by the FDA until the manufacturer’s voluntary removal of it from the market in 1983 [3]. In 1983 [4] and 1999 [5], the FDA determined that this drug combination was not withdrawn from sale for reasons of safety and effectiveness. In fact, its removal from the American market was temporally associated with a 2-fold increase in rates of hospitalization of pregnant women for the most severe form of NVP, hyperemesis gravidarum [6,7]. Over the last 3 decades a large body of evidence corroborated the fetal safety of this drug combination [8,9].

The rationale for the delayed-release characteristics of this combination is to allow women to take it before bedtime, when symptoms of NVP tend to be minimal, in order to counteract the increased symptoms more commonly experienced in the morning hours [10,11]. In April 2013, the FDA approved the sale of Diclegis®, an identical combination to the original Bendectin® and its Canadian equivalent Diclectin®, after a randomized blinded placebo- controlled trial [12]. The fetal safety of this medication has been proven by numerous studies. However, since it is the only FDA-approved medication for NVP, it is likely to be used by a large number of pregnant women, confirming maternal safety is an important public health question.

The objective of this study was to evaluate the maternal safety of Diclegis® in treating NVP compared to placebo.

## Methods

This is a secondary analysis of a double-blind, randomized, multicenter, placebo-controlled study of the delayed-release combination of doxylamine succinate (10 mg) and pyridoxine hydrochloride (10 mg) (Diclegis®) in the treatment of NVP [12]. The study was approved by the IRB of the University of Texas, Galveston, University of Pittsburg and Georgetown University. The subjects were pregnant, at least 18 years of age, in the gestational age range of 7–14 weeks, suffering from NVP, had a PUQE score ≥ 6) [13-15], and had not responded to conservative management consisting of dietary/lifestyle advice [16]. Women treated by other antiemetics, suffering from chronic medical conditions, or those who could not communicate in either English or Spanish, were excluded. After physical examination and laboratory tests (hemoglobin and blood count, liver function tests, electrolytes, amylase), and after confirming *in utero* singleton pregnancies by ultrasound, women were randomized, using a computerized program, to receive Diclegis® (doxylamine succinate 10 mg and pyridoxine hydrochloride 10 mg) or placebo of similar look. Two tablets of study drug (Diclegis® or placebo) were administered at bedtime on Day 1. If symptoms of nausea and vomiting persisted into the afternoon hours of Day 2 (*i.e.,* PUQE Score above 3), the subject was directed to take her usual dose of 2 tablets at bedtime and an additional tablet the next morning on Day 3. Based upon assessment in the clinic on Day 4 (+/−1 day), the subject might have been directed to take an additional 4th tablet mid-afternoon to control evening symptoms. The minimum assigned study medication was 2 tablets daily at bedtime, increasing when indicated to the maximal dosage of 4 tablets per day according to the timing, duration, severity, and frequency of the symptoms experienced by the subject. The study had a 15 day period consisting of 14 dosing days. Telephone contact was made on day 2, 6, 12, and 14 in order to assess subject diary information, adverse events (AEs), concomitant medications, and compliance with the study medication. Patients returned to the clinic in the morning prior to their morning dose on Day 4 (+/− 1 day), Day 8 (+/− 1 day), and on Day 15 (+/− 1 day; end of study visit) to collect diary report and complete all study related data.

Subjects completed the Pregnancy-Unique Quantification of Emesis (PUQE) score and the study diary (once daily every morning prior to study dose at approximately the same time each day). They also completed the Global Assessment of Well Being scale of the PUQE on Days 1, 8, and 14 at the same time the PUQE score was completed.

Adverse events and concomitant medications were recorded at all visits and follow-up phone calls. An additional follow-up phone call was conducted 30 days after last dosing to capture serious adverse events for patients completing the treatment period or early termination.

The results of the effectiveness of the drug in this trial have been previously published [12].

The frequency and severity of all AEs were tabulated by treatment group, system organ class, preferred term severity, and relationship to study drug. In addition, laboratory tests were conducted on Days 1 and15 (±1 day).

Adverse events (AEs) experienced by the subjects that occurred on or after Day 1 (after the patient signed the informed consent form) through Day 15, were compared between groups using Pearson’s Chi-square test or Fisher’s exact test if more appropriate. The available sample size has 80% power to show a doubling in CNS depression with alpha of 5%.

## Results

Two hundred and eighty-three pregnant women experiencing NVP were enrolled and randomized. After written informed consent nine subjects randomized to Diclegis® (6.4%) and 18 randomized to Placebo (12.9%) withdrew consent. Therefore, 131 women in Diclegis® treated group and 125 receiving placebo were analyzed. The two groups did not differ in any demographic or medical characteristics (Table 1).

**Table 1:** **Comparison of demographic and medical characteristics of the two study groups in the ITT population**

	**Diclegis (n-131)**	**Placebo (n = 125)**	***P*** **-value**
**Ethnicity:**			0.48
**Hispanic or Latino**	53 (40.5%)	56 (44.8%)	
**Not Hispanic or Latino**	78 (59.5%)	69 (55.2%)	
Race:			0.59
Asian	2 (1.5%)	1 (0.8%)	
Black or African American	49 (37.4%)	48 (38.4%)	
White or Caucasian	80 (61.1%)	73 (58.4%)	
Unknown		3 (2.4%)	
Previous Pregnancy	101 (77.1%)	94 (75.2%)	0.64
Smoking during pregnancy	17 (13.0%)	16 (12.8%)	0.97
Maternal Age (yr)	25.9 ± 6	25.0 ± 5.7	0.23
Weight (kg)	74.10 ± 22.30	75.91 ± 22.19	0.50
(lbs)	163.35 ± 49.17	167.34 ± 48.91	0.50
BMI (kg/m^2^):			0.42
Underweight	5 (3.8%)	4 (3.2%)	
Normal	39 (29.8%)	38 (30.4%)	
Overweight	31 (23.7%)	40 (32.0%)	
Obese	55 (42.0%)	42 (33.6%)	
BMI (kg/m^2^)			0.95
Mean ± SD	28.77 ± 7.60	29.67 ± 11.20	
Median	27.97	26.83	
Gestational age at start of NVP (weeks)			0.90
Mean ± SD	5.5 ± 1.8	5.4 ± 1.7	
Gestational age at enrollment (weeks)			0.75
Mean ± SD	9.3 ± 2.0	9.3 ± 1.8	
PUQE score at Enrollment			0.44
Mean ± SD	9.0 ± 2.1	8.8 ± 2.1	
Median	9.0	8.0	
Global Assessment of Well Being			
Mean ± SD	5.0 ± 2.3	5.4 ± 2.2	
Median	5.0	5.0	

The use of Diclegis® was not associated with an overall increased rate of adverse effects as compared to the Placebo group (Table 2). Of particular interest, the active drug was not associated with increased rates of symptoms known to be associated with antihistamines, such as sedation, symptoms of CNS depression and gastrointestinal or anticholinergic symptoms (Table 3). Diclegis® was also not associated with either more frequently occurring adverse events (Table 4), or with an increase in Treatment Emergent Adverse Events (TEAEs) -as defined by the investigators, blinded to study drug allocation (Table 5). TEAEs were defined as any adverse event that emerged after the treatment (either the drug or the placebo) were commenced.

**Table 2:** **Overall summary of tolerability/adverse events for ITT-S subjects**

	**Treatment group**		
	**Diclegis (N = 131)**	**Placebo (N = 127)**	**P-value** ^**1**^
**Measure of tolerability**			
Number of Subjects with at least one treatment-emergent AE	74 (56.5%)	65 (51.2%)	0.393
Number of Subjects with a serious treatment-emergent AE	4 (3.1%)	4 (3.1%)	1.000^2^
Number of Subjects with at least one Related AE	40 (30.5%)	32 (25.2%)	0.339
Number of Subjects discontinuing study drug due to AE	6 (4.6%)	4 (3.1%)	0.749^2^
Number of deaths	0	0	_
Overall treatment0emergent AEs			
Number of Subjects with at least one Mild AE	62 (47.3%)	59 (46.5%)	0.221
Number of Subjects with at least one Moderate AE	5 (3.8%)	1 (0.8%)	0.215^2^
Number of Subjects with at least one Severe AE	7 (5.3%)	5 (3.9%)	0.711
Number of Subjects with Unrelated AE	34 (26.0%)	33 (26.0%)	0.570
Number of Subjects with at least one Possibly Related AE	24 (18.3%)	23 (18.1%)	0.714
Number of Subjects with at least one Probably Related AE	13 (9.9%)	8 (6.3%)	0.388
Number of Subjects with at least on Definitely Related AE	3 (2.3%	1 (0.8%)	0.623^2^

^1^The p-value for comparing Treatment groups uses Chi-square test method.

^2^P-value is calculated using Fisher’s exact test method.

Related category includes Possible, Probable, and Definite relationships. Unrelated category includes unlikely and not related.

Subjects reporting more than one AE will only be counted under the strongest relationship and/or severity.

Mild: asymptomatic or mild symptoms, intervention not needed; Moderate: minimal, local or non invasive intervention indicated; Severe: medically significant.

**Table 3:** **Treatment Emergent Adverse Events (TEAEs) in the study for ITT-S subjects**

	**Treatment group**		
**System Organ Class (SOC) preferred term**	**Diclegis (N = 131)**	**Placebo (N = 127)**	**P-value** ^**1**^
# of Subjects with at least one TEAE	74 (56.5%)	65 (51.2%)	0.39
Cardiac disorders	1 (0.8%)	1 (0.8%)	1.000^2^
Palpitations	1 (0.8%)	1 (0.8%)	1.000^2^
Eye disorders	1 (0.8%)	0	1.000^2^
Dry eye	1 (0.8%)	0	1.000^2^
Gastrointestinal disorders	23 (17.6%)	22 (17.3%)	0.960
Constipation	2 (1.5%)	2 (1.6%)	1.000^2^
Dry mouth	4 (3.1%)	1 (0.8%)	0.370^2^
Haematemesis	0	1 (0.8%)	0.492^2^
Feeling jittery	1 (0.8%)	0	1.000^2^
**Laboratory Investigations**	7 (5.3%)	6 (4.7%)	0.820
Alanine aminotransferase increased	0	1 (0.8%)	0.492^2^
Aspartate aminotransferase increased	0		
Blood albumin decreased	1 (0.8%)	0	1.000^2^
Blood amylase increased	2 (1.5%)	2 (1.6%)	1.000^2^
Blood chloride decreased	0	1 (0.8%)	0.492^2^
Blood creatinine increased	1 (0.8%)	1 (0.8%)	1.000^2^
Blood lactate dehydrogenase increased	1 (0.8%)	0	1.000^2^
Blood sodium decreased	0	1 (0.8%)	0.492^2^
Blood triglycerides increased		1 (0.8%)	0
Gamma-glutamyltransferase increased	1 (0.8%)	1 (0.8%)	1.000^2^
Heart rate increased	0	1 (0.8%)	0.492^2^
Platelet count decreased	1 (0.8%)	0	1.000^2^
**Nervous system disorders**	42 (32.1%)	37 (29.1%)	0.610
Dizziness	8 (6.1%)	8 (6.3%)	0.949
Headache	17 (13.0%)	20 (15.7%)	0.526
Loss of consciousness	0	1 (0.8%)	0.492^2^
Poor quality sleep	1 (0.8%)	0	1.000^2^
Somnolence	19 (14.5%)	15 (11.8%)	0.523
Syncope	1 (0.8%)	1 (0.8%)	1.000^2^
Fatigue	9 (6.9%)	8 (6.3%)	0.853

^1^The p-value for comparing Treatment groups uses Chi-square test method.

^2^P-value is calculated using Fisher’s exact test method.

At each level of summarization (SOC/preferred term), subjects reporting more than one AE will only be counted once.

**Table 4:** **Most frequently occurring Treatment Emergent Adverse Events (TEAEs) in the study for ITT-S subjects**

	**Treatment group**		
**System Organ Class (SOC) preferred term**	**Diclegis (N = 131)**	**Placebo (N = 127)**	**P-value** ^**1**^
# of Subjects with at least one TEAE	74 (56.5%)	65 (51.2%)	0.393
Gastrointestinal disorders	23 (17.6%)	22 (17.3%)	0.960
Abdominal pain	5 (3.8%)	8 (6.3%)	0.362
General disorders and administration site	13 (9.9%)	12 (9.4%)	0.897
Conditions			
Fatigue	9 (6.9%)	8 (6.3%)	0.949
Musculoskeletal and connective tissue	11 (8.4%)	4 (3.1%)	0.072
Disorders			
Back pain	7 (5.3%)	4 (3.1%)	0.383
Nervous system disorders	42 (32.1%)	37 (29.1%)	0.610
Dizziness	8 (6.1%)	8 (6.3%)	0.949
Headache	17 (13.0%)	20 (15.7%)	0.526
Somnolence	19 (14.5%)	15 (11.8%)	0.523

^1^The p-value for comparing Treatment groups uses Chi-square test method.

TEAEs that are considered most frequently occurring include the events (in preferred terms) reported by at least 5% of subjects in any of the treatment groups.

At each level of summarization (SOC/preferred term), subjects reporting more than one AE will only be counted once.

**Table 5:** **Treatment Emergent Adverse Events (TEAEs) with respect to relationship to study drug- related vs. unrelated for ITT-S subjects**

	**Treatment group**			
	**Diclegis (N = 131)**		**Placebo (N = 127)**	
**System Organ Class (SOC) preferred term**	**Related**	**Unrelated**	**Related**	**Unrelated**
# of Subjects with at least one	40 (30.5%)	34 (26.0%)	32 (25.2%)	33 (26.0%)
TEAE in the study				
Cardiac disorders	1 (0.8%)	0	0	1 (0.8%)
Palpitations	1 (0.8%)	0	0	1 (0.8%)
Eye disorders	0	1 (0.8%)	0	0
Dry eye	0	1 (0.8%)	0	0
Gastrointestinal disorders	8 (6.1%)	15 (11.5%)	8 (6.3%)	14 (11.0%)
Abdominal pain	1 (0.8%)	4 (3.1%)	3 (2.4%)	5 (3.9%)
Abdominal pain upper	0	3 (2.3%)	2 (1.6%)	3 (2.4%)
Constipation	1 (0.8%)	1 (0.8%)	1 (0.8%)	1 (0.8%)
Diarrhea	2 (1.5%)	2 (1.5%)	1 (0.8%)	1 (0.8%)
Dry mouth	4 (3.1%)	0	1 (0.8%)	0
Dyspepsia	1 (0.8%)	4 (3.1%)	1 (0.8%)	1 (0.8%)
Flatulence	0	0	0	1 (0.8%)
Salivary hypersecretion	0	0	0	1 (0.8%)
General disorders and administration	7 (5.3%)	6 (4.6%)	6 (4.7%)	6 (4.7%)
Feeling jittery	1 (0.8%)	0	0	0
Nervous system disorders	33 (25.2%)	9 (6.9%)	24 (18.9%)	13 (10.2%)
Dizziness	6 (4.6%)	2 (1.5%)	5 (3.9%)	3 (2.4%)
Headache	8 (6.1%)	9 (6.9%)	8 (6.3%)	12 (9.4%)
Loss of consciousness	0	0	0	1 (0.8%)
Poor quality sleep	0	1 (0.8%)	0	0
Somnolence	19 (14.5%)	0	15 (11.8%)	0
Syncope	1 (0.8%)	0	0	1 (0.8%)
Fatigue	6 (4.6%)	3 (2.3%)	5 (3.9%)	3 (2.4%)

Related category includes Possible, Probable, and Definite relationships. Unrelated category includes unlikely and not related.

At each level of summarization (SOC/preferred term), subjects reporting more than on AE will only be counted once under the strongest relationship.

## Discussion

The randomized trial described herein has previously shown the superiority of Diclegis® over placebo in treating the symptoms of NVP in American women managed in 3 academic centers [12].

In Canada, the doxylamine succinate-pyridoxine hydrochloride combination (Diclectin®) has been available since 1979 [17], with a large number of studies corroborating the initial FDA evaluation of its efficacy and safety [18-20]. The present study had a placebo arm, as symptoms of NVP tend to subside spontaneously in most women by the end of the first trimester [1]. In addition, when considering maternal safety associated with the first trimester of pregnancy, symptoms such as fatigue, tendency to sleep and dehydration, may be erroneously attributed to the drug, rather than to both pregnancy and NVP.

The use of Diclegis® was not associated with an increased risk of any adverse effects when compared to placebo, lending important reassurance to its use by large numbers of pregnant women.

It may be puzzling, however, how symptoms of CNS depression, so typical of the sedating antihistamines, were more prevalent in the active arm of the study. The answer may lie in the demographics of the study population as presented in Table 1.

The mean weight of the women in the study was 75 kg (165 lb), rendering 24% of them overweight and 42% obese. When the original Bendectin® studies were conducted the mean weight of participating women was 20 kg lower on average. As a result we might hypothesize that the relatively lower weight-adjusted dose given to our mostly overweight and obese study participants could have mitigated adverse drug effects.

A previous study determined the incidence of adverse maternal effects among 225 women taking Diclectin® at the recommended (n = 123) or higher than recommended (n = 102) doses [21]. One-third (33.6%) of those women reported having adverse CNS effects (sleepiness, tiredness, and/or drowsiness) temporally related to the medication, a rate very similar to the present study (28.3%). In that study there was no association between the dose per kg and rates of reported maternal adverse effects with doses ranging from 0.1 mg/kg to 2.0 mg/kg (1–12 tablets). In addition, the higher than standard dose of Diclectin®, when calculated per kg of body weight, did not affect either the incidence or severity of maternal adverse effects. Similarly, no excess was recorded for other typical adverse effects of antihistamine, such as those involved in low gastrointestinal motility or anticholinergic effects (e.g. dry mouth, dysrhythmia).

In a recent population- based Canadian study, pregnant women in the first trimester of pregnancy, when NVP and the use of Diclegis® is at its peak, did not have a higher risk of car crushes, whereas there was a 46% increased risk in the second trimester, when most morning sickness has subsided and the drug is not used [21]. This may serve as a population-based corroboration of the present results, showing that Diclegis® is not associated with measurable CNS depression.

## Conclusions

Based on this secondary analysis of results from a double blind placebo controlled trial, the pyridoxine-doxylamine combination appears to be safe to the pregnant woman suffering from NVP.

CTR No. NCT006 14445, 2007.
